# Activated TRPA1 plays a therapeutic role in TMZ resistance in glioblastoma by altering mitochondrial dynamics

**DOI:** 10.1186/s12860-022-00438-1

**Published:** 2022-08-19

**Authors:** Hao Chen, Chunlin Li, Haiyang Hu, Bin Zhang

**Affiliations:** 1 Department of Neurosurgery, Affiliated Hospital of Jining Medical University, Jining, China; 2 Department of Vascular Surgery, Shanghai Jiao Tong University Affiliated Sixth People’s Hospital, 600 Yishan Road, Shanghai, 200233 China; 3 Trauma Center, Shanghai General Hospital, Shanghai Jiaotong University School of Medicine, Shanghai, 201620 China

**Keywords:** Glioblastoma, Temozolomide, TRPA1, Oxidative stress, Mitochondrial dysfunction

## Abstract

**Background:**

Glioblastoma (GBM) represents nearly one-half of primary brain tumors, and the median survival of patients with GBM is only 14.6 months. Surgery followed by radiation with concomitant temozolomide (TMZ) therapy is currently the standard of care. However, an increasing body of evidence suggests that GBM acquires resistance to TMZ, compromising the effect of the drug. Thus, further exploration into the mechanism underlying this resistance is urgently needed. Studies have demonstrated that TMZ resistance is associated with DNA damage, followed by altered reactive oxygen species (ROS) production in mitochondria. Studies have also showed that Ca^2+^-related transient receptor potential (TRP) channels participate in GBM cell proliferation and metastasis, but the detailed mechanism of their involvement remain to be studied. The present study demonstrates the role played by TRPA1 in TMZ resistance in GBM and elucidates the mechanism of resistance.

**Methods:**

U251 and SHG-44 cells were analyzed in vitro. A CCK-8 assay was performed to verify the effect of TMZ toxicity on GBM cells. Intracellular ROS levels were detected by DCFH-DA assay. A MitoSOX Red assay was performed to determine the mitochondrial ROS levels. Intracellular Ca^2+^ levels in the cells were determined with a Fluo-4 AM calcium assay kit. Intracellular GSH levels were determined with GSH and GSSG Assay Kit. MGMT protein, Mitochondrial fission- and fusion-, apoptosis- and motility-related protein expression was detected by western blot assay. A recombinant lentiviral vector was used to infect human U251 cells to overexpress shRNA and generate TRPA1^+/+^ and negative control cells. All experiments were repeated.

**Results:**

In the U251 and SHG-44 cells, TMZ induced a small increase in the apoptosis rate and intracellular and mitochondrial ROS levels. The expression of antioxidant genes and antioxidants in these cells was also increased by TMZ. However, pretreatment with a TRPA1 agonist significantly decreased the level of antioxidant gene and antioxidants expression and enhanced intracellular and mitochondrial ROS levels. Also TMZ induced the level of MGMT protein increased, and pretreatment with a TRPA1 agonist decreased the MGMT expression. Moreover, Ca^2+^ influx, mitochondrial damage and cell apoptosis were promoted, and the balance between mitochondrial fission and fusion protein expression was disrupted in these GBM cells. Pretreatment with a TRPA1 inhibitor slightly enhanced the level of antioxidant gene expression and reduced the apoptosis rate. TRPA1 gene overexpression in the U251 cells was similar to that after inhibitor intervention, confirming the aforementioned experimental results.

**Conclusion:**

The present study proved that activating TRPA1 in glioma cells, which leads to mitochondrial damage and dysfunction and ultimately to apoptosis, may decrease the TMZ resistance of GBM cells.

**Supplementary Information:**

The online version contains supplementary material available at 10.1186/s12860-022-00438-1.

## Introduction

Glioblastoma (GBM), with an astrocytic lineage, is the most familiar primary malignant tumor [1]. It represents > 45% of primary brain tumors and has an incidence rate of 3.2 cases/100,000 people per year [2]. The median survival of GBM patients is only 14.6 months, and after diagnosis, only 3% of patients live longer than 5 years [3, 4]. Moreover, the therapeutic agents available for GBM treatment are limited. The blood–brain barrier presents an obstacle to drug penetration. In addition, the tendency for the tumor to develop resistance is the main impediment to therapeutic development. As the standard of care, surgery with maximal tumor resection followed by external-beam radiation with concomitant temozolomide (TMZ) therapy is applied. The main efficacy of TMZ is to methylate the O6 residues of guanine so as to prevent DNA duplication during cell proliferation and to induce cell apoptosis [5]. The chemoresistance caused by the intracellular activity of O6-methylguanine DNA-methyltransferase (MGMT), a DNA repair enzyme reversing the anti-tumor effect of TMZ by specifically removing the methyl group from O6 -positions of guanine residues [6]. However, an increasing number of studies have demonstrated that GBM cells acquire resistance to TMZ [2]. The drug acts through the induction of lethal DNA damage and subsequent production of radical oxygen species (ROS) [7], but the treatment provides only short-term results, and as many as 90% of patients who undergo surgical resection can expect disease recurrence [3]. Furthermore, the mechanisms through which cells acquire resistance is complicated, and multifactorial mechanisms are involved in TMZ resistance [8, 9]. Therefore, it is necessary to explore the specific mechanism of TMZ resistance and novel therapeutic strategies that target TMZ resistance [10].

Due to their adaptation mechanisms in sustaining tumorigenesis, cancer cells normally show elevated basal intracellular ROS levels without harmful consequences. However, excessive oxidative stress can overcome these ROS increases and restore cancer cells vulnerability to ROS-mediated damage, further improving the therapeutic response [11–13]. Resistance to DNA-damaging agents, including TMZ, is usually followed by altered ROS production in mitochondria [14, 15], which are the major ROS-producing organelles. ROS are mainly generated by mitochondria as electrons leak from electron transport chains, which leads to partial oxygen reduction and superoxide formation [16]. Mitochondria are subcellular organelles with double membranes. The functions of mitochondria include regulating the cellular redox state and signaling, cell apoptosis and proliferation [17]. The structure and function of mitochondria are regulated by a fission/fusion process [18]. An imbalance in mitochondrial fission and fusion induces organelle structural damage and decreased oxidative phosphorylation [19]. Mitochondrial fusion is regulated by the fusion proteins optic atrophy 1 (OPA1) and mitofusin 1 and 2 (MFN1 and MFN2). Mitochondrial fission is mediated by fission proteins, including dynamin-related protein 1 (DRP1) [19]. We speculated that disrupted mitochondrial dynamics may play a key role in TMZ resistance in GBM.

Many studies have proven that transient receptor potential (TRP) channels regulate the structure and function of mitochondria [20]. Various human TRP channels, which are encoded by genes in multiple families, can modulate cell function by triggering a transient increase in the intracellular Ca^2+^ concentration [21]. Although the fundamental biochemical capabilities of TRP channels are well understood, their functions in specialized cells [22] and possible importance of channelopathies remain unclear [23]. Among these channels, TRPA1 plays an important role in the inflammatory response and tissue damage. TRPA1 is recognized as a “gatekeeper of inflammation” [24] and as a calcium-permeable and ROS-sensitive cation channel [25, 26]. TRPA1 can be activated in neurons and cancer cells by various stimuli, including ROS [27, 28]. Upon activation of TRPA1 in cancer cells, increases in Ca^2+^ influx modulates cell migration and, presumably, tumor invasion [29]. Ca^2+^-dependent antiapoptotic pathways can be activated by TRPA1 without affecting the cellular redox status [30].

Hence, in the present study, we hypothesized that activating TRPA1 in glioma cells, which leads to mitochondrial damage and dysfunction and ultimately to cell apoptosis, may decrease the TMZ resistance of GBM cells. The objectives of our study were to 1) ascertain the effect of TRPA1 regulation on the apoptosis, oxidative stress and mitochondrial function of TMZ-treated GBM cells and 2) explore the mechanism of TRPA1 to propose promising new therapeutic strategies for GBM treatment.

## Materials and methods

### Cell culture and drug treatment

U251 cells and SHG-44 cells (Shanghai Institutes for Biological Sciences, China Academy of Science, Shanghai) were cultured in high-glucose Dulbecco’s modified Eagle’s medium (HyClone, Logan, UT, USA) with 10% fetal bovine serum (BioInd, Kibbutz Beit Haemek, Israel), 100 U/ml penicillin and 100 μg/ml streptomycin (Thermo Fisher Scientific, Waltham, MA, USA) at 37 °C in 5% CO_2_.

On the basis of observations in a preliminary study, U251 cells and SHG-44 cells were treated with 100 μΜ temozolomide (MedChemExpress, MCE, New Jersey, USA) for 24 h to cause cell injury. The cells were pretreated with 100 μM TRPA1 agonist (PF-4840154, MCE) or 100 μM TRPA1 antagonist (HC030031, MCE) for 1 h and then cultured with vehicle (culture medium) or TMZ for another 24 h.

### Cell viability assay

Cell Counting Kit-8 (CCK-8; Dojindo, Kumamoto, Japan) assays were performed to measure the effects of the vehicle, TMZ, and a TRPA1 agonist and inhibitor on cell proliferation. Briefly, cells were cultured at a density of 5 × 10^4^ cells/well in a 96-well plate, incubated overnight at 37 °C, and then treated with vehicle or other irritants. The treated cells were incubated for 24 h and washed with PBS. Then, following the CCK-8 kit manufacturer’s protocol, the cells were incubated with the working solution of CCK-8 for 2 h at 37 °C. The absorbance was measured at 450 nm with a iMark microplate reader (Molecular Devices, Sunnyvale, USA).

### Measurement of intracellular ROS and mitochondrial ROS levels

DCFH-DA (D6883, Sigma–Aldrich, St. Louis, MO, USA) was used to detect intracellular ROS generation through fluorescence microscopy with a fluorescence plate reader. Briefly, in each group, cells were seeded at a density of 5 × 10^4^ in 96-well black plates in 6 parallel wells. After 24 h, the cells were stained with 10 μM DCFH-DA at 37 °C for 15 min in the dark. Then, every well was washed three times with PBS, and the level of intracellular ROS was determined by microscopy (Leica, Germany) or by a Flexstation®2 fluorescence plate reader (Molecular Devices, San Jose, CA, USA) at excitation and emission wavelengths of 488 nm and 525 nm, respectively.

The activity levels of the mitochondrial ROS in cells were measured by MitoSOX Red (Invitrogen) assay, a redox-sensitive fluorescent probe that targets mitochondria in cells. In each group, cells were seeded at a density of 5 × 10^4^ in 96-well black plates with 6 parallel wells. After 24 h, the cells were stained with 5 mmol/ml MitoSOX Red probe at 37 °C in the dark for 10 mins. Then, every well was washed twice with PBS, and red fluorescence was detected with a Flexstation®2 fluorescence plate reader at excitation and emission wavelengths of 510 nm and 580 nm, respectively.

### Measurement of Ca2+ influx

Intracellular Ca^2+^ levels in the cells were determined according to the manufacturer’s instructions for a Fluo-4 AM calcium assay kit (Beyotime, Jiangsu, China). Briefly, in each group, the cells were seeded at a density of 5 × 10^4^ in 96-well black plates with 6 parallel wells. The experimental groups were pretreated with agonist or antagonist for 1 h, and then, all groups were exposed to TMZ for 2 mins. The cells were washed three times and stained with 5 μM Fluo-4 AM at 37 °C in the dark for 30 mins. Then, the cells in every well were washed three times with PBS, and the level of fluorescence was determined at excitation and emission wavelengths of 488 nm and 516 nm, respectively, by microscopy (Leica) with a Flexstation®2 fluorescence plate reader.

### Western blot (WB) assay

The expression levels of DRP1, MFF, OPA1, MFN2, BAX, BCL2, TRPA1 and GAPDH were detected by WB assay. Total cell proteins in every group were extracted with RIPA buffer (Beyotime). Protein concentrations were determined with a BCA protein assay kit (Beyotime). Total protein samples were separated by 10% SDS–PAGE, and then, the proteins were transferred to a PVDF membrane. Finally, 5% nonfat milk dissolved in Tris-buffered saline with Tween 20 (TBST, BioTNT, Shanghai, China) was used to block the membrane for 4 h. The membrane was tailored according to the molecular weight of the target protein, then the cropped membrane was incubated with primary antibodies against DRP1, MFF, OPA1, MFN2, BAX, BCL2, TRPA1, MGMT, Cleaved Caspase-3, Caspase-3 and GAPDH (all diluted 1:1000, Cell Signaling Technology, Boston, MA, USA) overnight at 4 °C. Horseradish peroxidase (HRP)-conjugated goat anti-rabbit IgG (1:15000, Cell Signaling Technology) and secondary antibodies were incubated with the membrane at room temperature for 2 h. Then, the membrane was washed 3 times in TBST and visualized with an enhanced chemiluminescent (ECL) detection system.

### Reverse transcription–polymerase chain reaction (RT–PCR)

RT–PCR was used to test the mRNA levels of antioxidants (MnSOD, HO-1 and NQO1). Total RNA was extracted from cells with TRIzol reagent (TaKaRa, Dalian, Liaoning, China). The concentration of the total RNA was determined with an ultraviolet spectrophotometer. The Prime Script™ RT Master Mix Kit (TaKaRa) was used to conduct reverse transcription according to the manufacturer’s instructions. RT–PCR was performed by Power Green qPCR Mix (TaKaRa) and the ABI ViiATM 7 System. The specific primers (Table 1) for MnSOD, HO-1, NQO1 and β-actin were generated by BioTNT (Shanghai, China). All samples were assayed in triplicate, and the values were normalized to the level of β-actin.

**Table 1:** The sequences of forward and reverse primers of antioxidants (HO-1, NQO1 and MnSOD) and β-actin

Forward	Reverse
(HO)-1 CTGCCCAAACCACTTCTGTT	ATAAGAAGGCCTCGGTGGAT
NQO1 CAGTGGCATGCACCCAGGGAA	GCATGCCCCTTTTAGCCTTGGCA
MnSOD ACAGGCCTTATTCCACTGCT	CAGCATAACGATCGTGGTTT
β-actin ACCGAGCGCGGCTACA	CAGCCGTGGCCATCTCTT

### Measurement of GSH levels

Intracellular glutathione (GSH) levels in the cells were determined according to the manufacturer’s instructions for a GSH and GSSG Assay Kit (Beyotime, Jiangsu, China). Briefly, in each group, the cells were seeded at a density of 1 × 10^6^ in 6-well plates with 6 parallel wells. The experimental groups were pretreated with agonist or antagonist for 1 h, and then, all groups were exposed to TMZ for 24 h. The cells were washed with PBS. Then, supernatant of lysed cells was collected and tested as required, and the level of fluorescence was determined at wavelengths of 412 nm, by microscopy (Leica) with a Flexstation®2 fluorescence plate reader.

### Cell transduction with lentivirus

A recombinant lentiviral vector overexpressing short hairpin RNA (shRNA) was designed and constructed by Zorin (Shanghai, China). Human U251 cells were infected with 10^7^ TU/mL (multiplicity of infection [MOI] = 10) lentivirus-mediated shRNA (TRPA1^+/+^) or negative control shRNA (Con) for 12 h. The cells were collected to determine their interference efficiency by WB assay.

### Statistical analysis

Statistical analysis was performed with GraphPad Prism (version 7; GraphPad Software, Inc., San Diego, CA). One-way ANOVA with Bonferroni’s post hoc test (for equal variance) or Dunnett’s T3 post hoc test (for unequal variance) was performed for comparisons among multiple groups. *P* < 0.05 was considered to be statistically significant.

## Results

### Effects of TMZ and a TRPA1 agonist and inhibitor on GBM cell viability and intracellular ROS

**Fig. 1:**
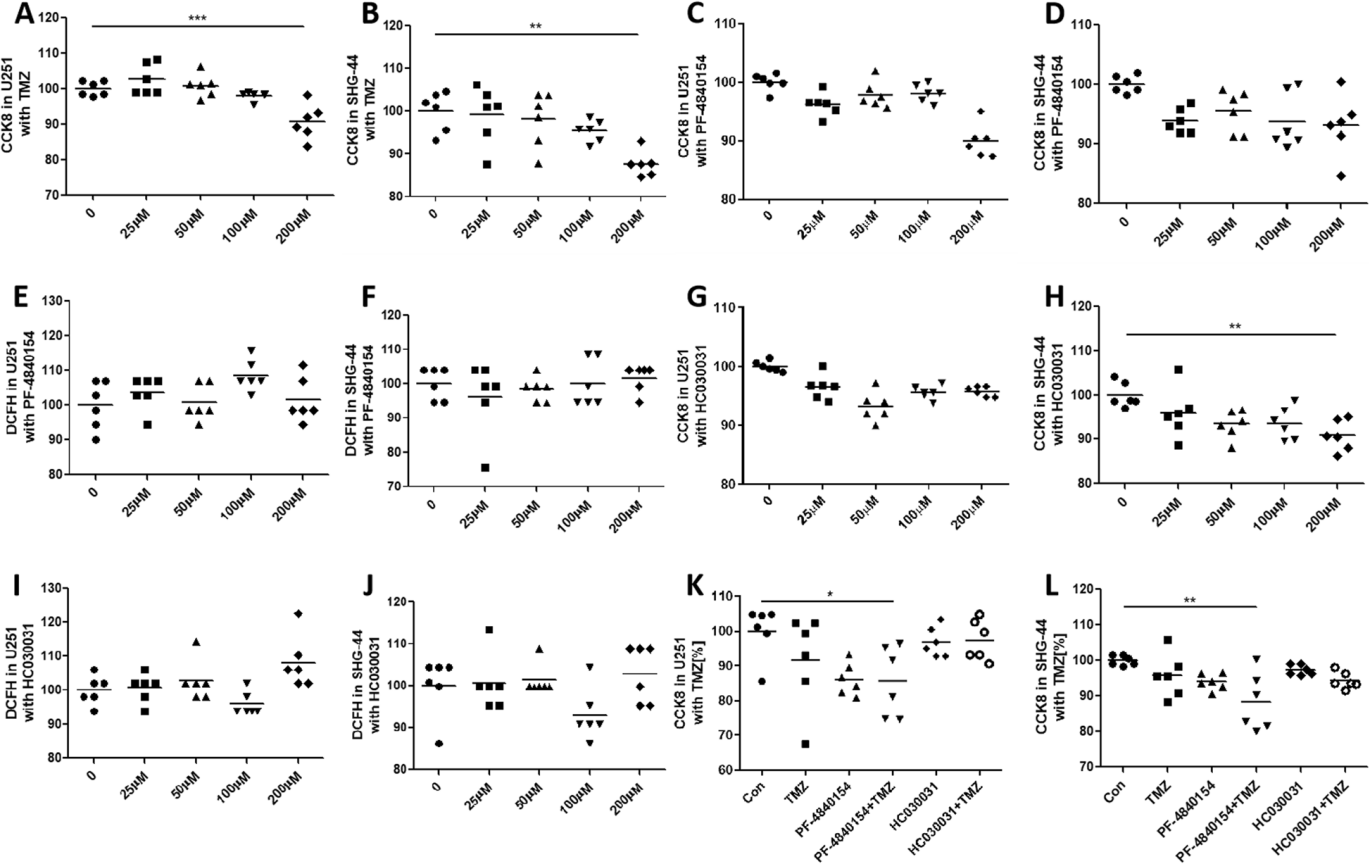
Effect of TMZ and a TRPA1 agonist and inhibitor on cell viability and intracellular ROS levels. TMZ (200 μΜ) significantly reduced the viability of U251 cells (**A**). No significant effect of TRPA1 agonist PF-4840154 or TRPA1 inhibitor HC030031 treatment on cell viability (**C**, **G**) or intracellular ROS levels (**E**, **I**) was observed in U251 cells at any treatment dose. Additionally, no significant effect of treatment with the TRPA1 agonist PF-4840154 or TRPA1 inhibitor HC030031 on cell viability (**B**, **D** and **H**) or intracellular ROS levels (**F**, **J**) in SHG-44 cells was observed at any dose. TMZ treatment or PF-4840154 and TMZ cotreatment decreased the viability of the U251 cells (**K**) and SHG-44 cells (**L**). *P < 0.05, **P < 0.01, ***P < 0.001,****P < 0.0001

To determine the optimum concentration for the all drugs used in the experiments, the influence of TMZ on GBM cell viability was tested by CCK-8 assay. As shown in Fig. 1A and B, 25 ~ 100 μM TMZ had no effect on the viability of the U251 and SHG-44 cells after 24 h of treatment. In addition, 200 μM TMZ significantly reduced cell viability compared to that of the control cells. Thus, 100 μM TMZ was used in the following experiments.

The effect of the TRPA1 agonist and inhibitor on GBM cell viability was also tested. Fig. 1C and D shows that 25 ~ 200 μM PF-4840154 had no effect on U251 or SHG-44 cell viability. Moreover, no significant reduction in intracellular ROS levels was observed in cells after treatment with 25 ~ 200 μM PF-4840154 for 24 h, as determined by DCFH-DA assay (Fig. 1E and F).

Similar results were observed when testing the effect of the TRPA1 inhibitor HC030031 on cell viability (Fig. 1G and H) and intracellular ROS levels (Fig. 1I and J), although 200 μM HC030031 reduced the viability of the SHG-44 cells.

Thus, 100 μM PF-4840154 and 100 μM HC030031 were used in the following experiments. We stimulated cells with the concentration of the drug that was experimentally obtained. In U281 cells, the concentration of TMZ treatment for 24 h did not significantly decrease cell viability. The TRPA1 agonist PF-4840154 had no effect on cell viability. However, pretreatment with PF-4840154 and subsequent TMZ administration reduced cell viability. Moreover, pretreatment with the TRPA1 inhibitor HC030031 showed no effect on cell viability (Fig. 1K). Similar results were found with SHG-44 cells. Cell viability was not influenced by TMZ, PF-4840154 or HC030031 exposure for 24 h, but pretreatment with PF-4840154 decreased cell viability after TMZ administration (Fig. 1L).

### Activating TRPA1 enhanced the effect of TMZ on cell apoptosis

To test whether TRPA1 or TMZ had a disruptive effect on cell apoptosis, we first measured the activity of the apoptosis-related proteins Caspase-3 and Caspase-9.

**Fig. 2:**
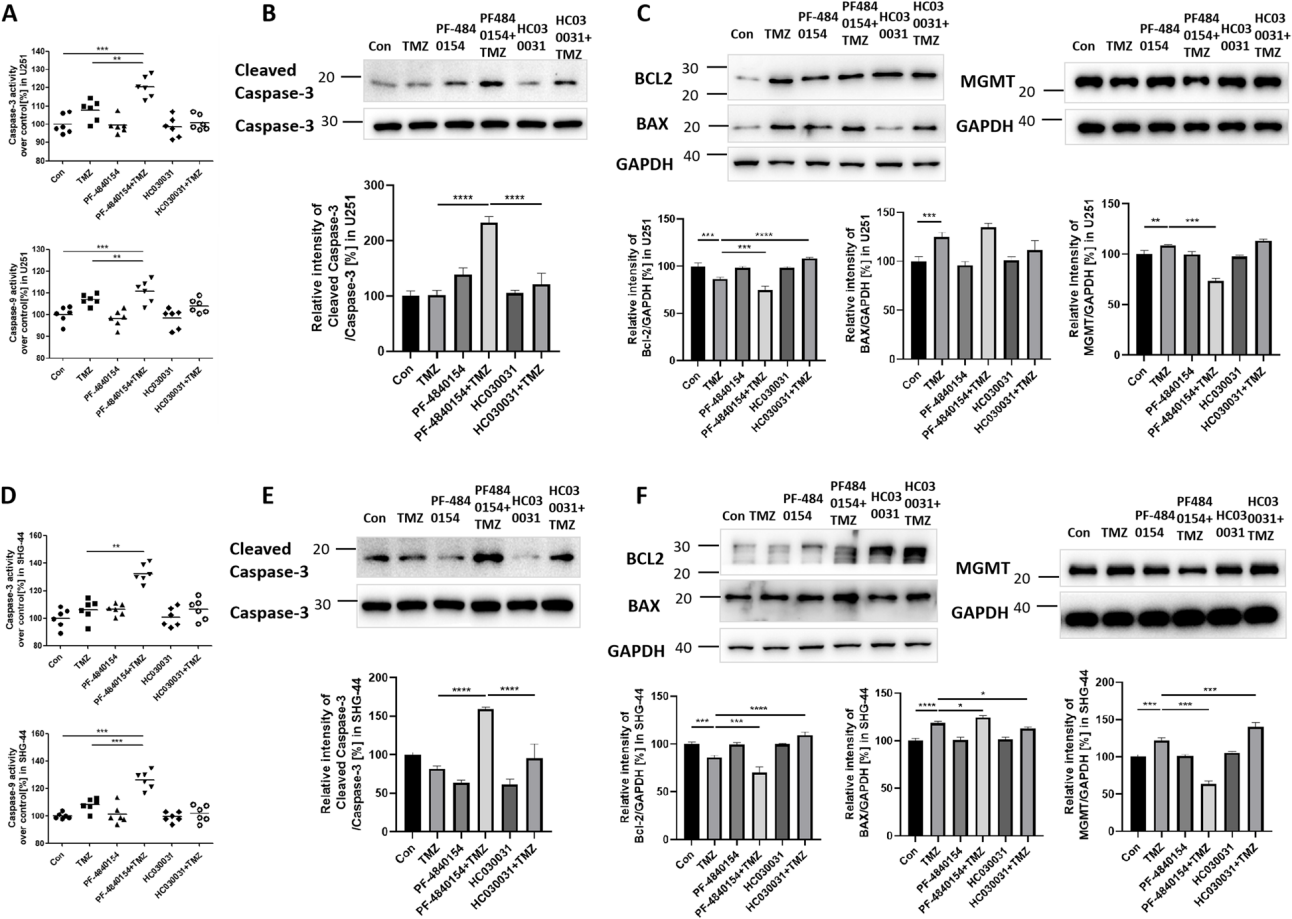
Effect of TMZ and a TRPA1 agonist and inhibitor on the apoptosis rate. PF-4840154 and TMZ cotreatment increased the activity of Caspase-3 and Caspase-9, and the protein expression of Cleaved Caspase-3 (**A**, **B**) in U251 cells compared to the control group and TMZ group. In the SHG-44 cells, the TMZ group and PF-4840154 and TMZ cotreatment group showed increased Caspase-3 activity and Cleaved Caspase-3 protein expression, and the HC030031 and TMZ cotreatment group showed decreased Cleaved Caspase-3 protein expression (**E**). PF-4840154 and TMZ cotreatment increased the activity of Caspase-3 and Caspase-9 (**D**) in the SHG-44 cells compared to the control and TMZ group. Western blotting was performed to detect the levels of Bcl-2 protein, BAX protein and MGMT protein after TMZ or drug treatment in U251 cells (**C**) and SHG-44 cells (**F**). GAPDH was used as an internal reference. Cropped photos of the blots are displayed, and the expression level was quantified by densitometry with ImageJ software. *P < 0.05, **P < 0.01, ***P < 0.001,****P < 0.0001

Pretreatment of U251 cells with PF-4840154 followed by TMZ administration significantly increased the activity of Caspase-3 and Caspase-9 compared to their activity in the TMZ-only treatment group (Fig. 2A). Pretreatment of the SHG-44 cells with PF-4840154 significantly increased the activity of Caspase-3 and Caspase-9 compared to the activity in the control group and TMZ group. In addition, pretreatment with HC030031 decreased the activity of Caspase-3 (Fig. 2D).

These results were verified by WB assay of the apoptosis-related proteins Cleaved Caspase-3, Bax and Bcl-2. Compared to TMZ groups, the level of Cleaved Caspase-3 elevated in PF-4840154 and TMZ cotreatment groups. However, pretreatment with HC030031 decreased Cleaved Caspase-3 expression, compared to pretreatment with PF-4840154 (Fig. 2B and E). The TMZ, PF-4840154 and cotreatment groups all showed elevated expression of the proapoptosis protein BAX and decreased levels of the anti-apoptosis protein Bcl-2. The expression of Bcl-2 was elevated in the HC030031 group (Fig. 2C and F). The same results were for both U251 and SHG-44 cells. Considering MGMT protein play the pivotal role in the determinant of TMZ resistance, we further tested the expression of MGMT in cells. In both U251 and SHG-44 cells, TMZ induced the MGMT protein increased. Pretreatment with PF-4840154 and subsequent TMZ administration reduced MGMT expression. Moreover, pretreatment with the TRPA1 inhibitor HC030031 showed MGMT expression more significant elevated compared with the TMZ group in SHG-44 cells (Fig. 2C and F). All original western blot images have been included in Supplementary original western blot images 1.

### Activating TRPA1 influenced the TMZ effects on cell viability and intracellular oxidative stress

**Fig. 3:**
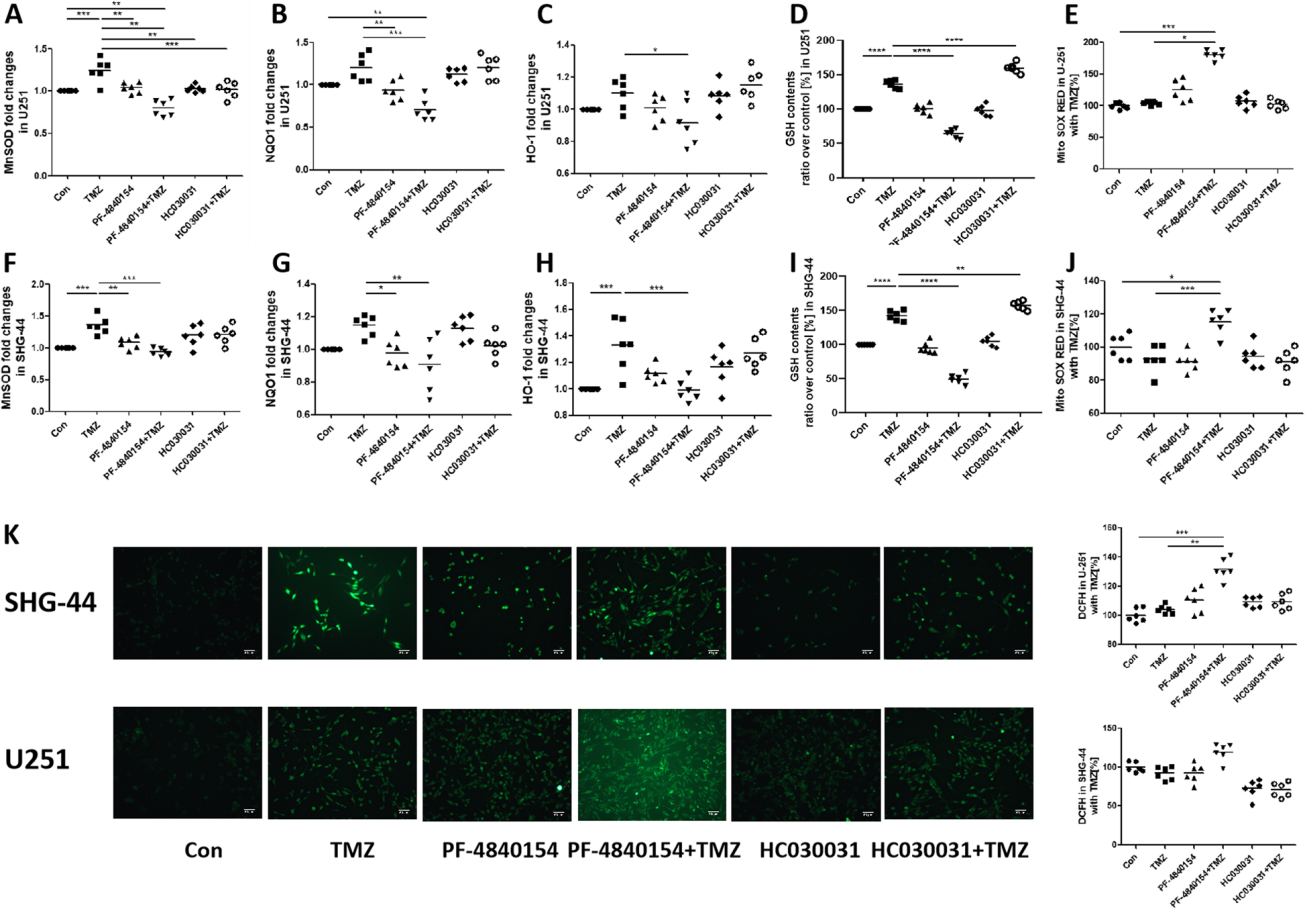
Effect of TMZ and a TRPA1 agonist and inhibitor on antioxidant gene expression. TMZ and PF-4840154 cotreatment prevented the effect of TMZ on antioxidant gene expression. Pretreatment with HC030031 or HC030031 and TMZ cotreatment significantly increased MnSOD mRNA expression. The fold change in mRNA expression of MnSOD (**A**, **F**), NQO1 (**B**, **G**), and heme oxygenase (HO)-1 (**C**, **H**) in U251 and SHG-44 cells as measured by quantitative RT–PCR. The expression of GSH were increased by exposure to TMZ and decreased in PF-4840154 and TMZ cotreatment group, as detected in U251 cells (**D**) and SHG-44 cells (**I**) with a fluorescence plate reader. The levels of mitochondrial ROS were increased by exposure to PF-4840154 and TMZ cotreatment, as detected by MitoSOX Red fluorescence in U251 cells (**E**) and SHG-44 cells (**J**) with a fluorescence plate reader. Intracellular ROS levels in in U251 cells and in SHG-44 cells were detected by DCFH-DA and fluorescence microscopy with a fluorescence plate reader (**K**). Representative microscopy images are shown (100 x) with mean values and data from individual plate reader experiments reported graphically below. *P < 0.05, **P < 0.01, ***P < 0.001, ****P < 0.0001

We hypothesized that GBM cell drug resistance is related to the oxidative stress resistance of tumor cells. Therefore, we evaluated the expression levels of the antioxidant genes MnSOD, NQO1 and HO-1, as well as antioxidants glutathione (GSH). TMZ exposure of U251 cells for 24 h significantly increased the mRNA expression of MnSOD compared with the basal level. NQO1 and HO-1 expression was also increased, although the differences were not statistically significant. Pretreatment with PF-4840154 significantly decreased the expression of MnSOD, NQO1 and HO-1 mRNA compared to that in the TMZ group (Fig. 3A, B and C). TMZ exposure of U251 cells for 24 h significantly increased the expression of GSH compared with the basal level. Pretreatment with PF-4840154 obviously decreased the expression of GSH compared to that in the TMZ group, while pretreatment with HC030031 reduced GSH level (Fig. 3D). Similar results were found with SHG-44 cells (Fig. 3F, G, H and I).

In addition to the detection of antioxidant genes, we intuitively noticed changes in mitochondrial ROS and intracellular ROS levels. The levels of mitochondrial ROS were measured by MitoSOX RED assay. U251 and SHG-44 cells exposure TMZ led to no change in the intracellular or mitochondrial ROS levels. Additionally, treatment with only the TRPA1 agonist PF-4840154 exerted no effect on ROS production, while treatment with PF-4840154 and TMZ significantly increased mitochondrial ROS levels (Fig. 3E and J). Representative pictures are showing the intracellular ROS levels in the U251 and SHG-44 cells, detected by the DCFH-DA assay, are shown in Fig. 3K. The results showed that PF-4840154 and TMZ synergistically increased the intracellular ROS levels significantly and that HC030031 reduced the intracellular ROS levels. The quantitative results are shown on the right. Moreover, pretreatment with the TRPA1 inhibitor HC030031 slightly reduced intracellular and mitochondrial ROS levels, although the differences were not statistically significant (Fig. 3K).

Our results indicate that the increase in intracellular TRPA1 levels can significantly increase the ability of TMZ to damage tumor cells through ROS generation.

### Activating TRPA1 and TMZ treatment increased intracellular Ca2+ levels

**Fig. 4:**
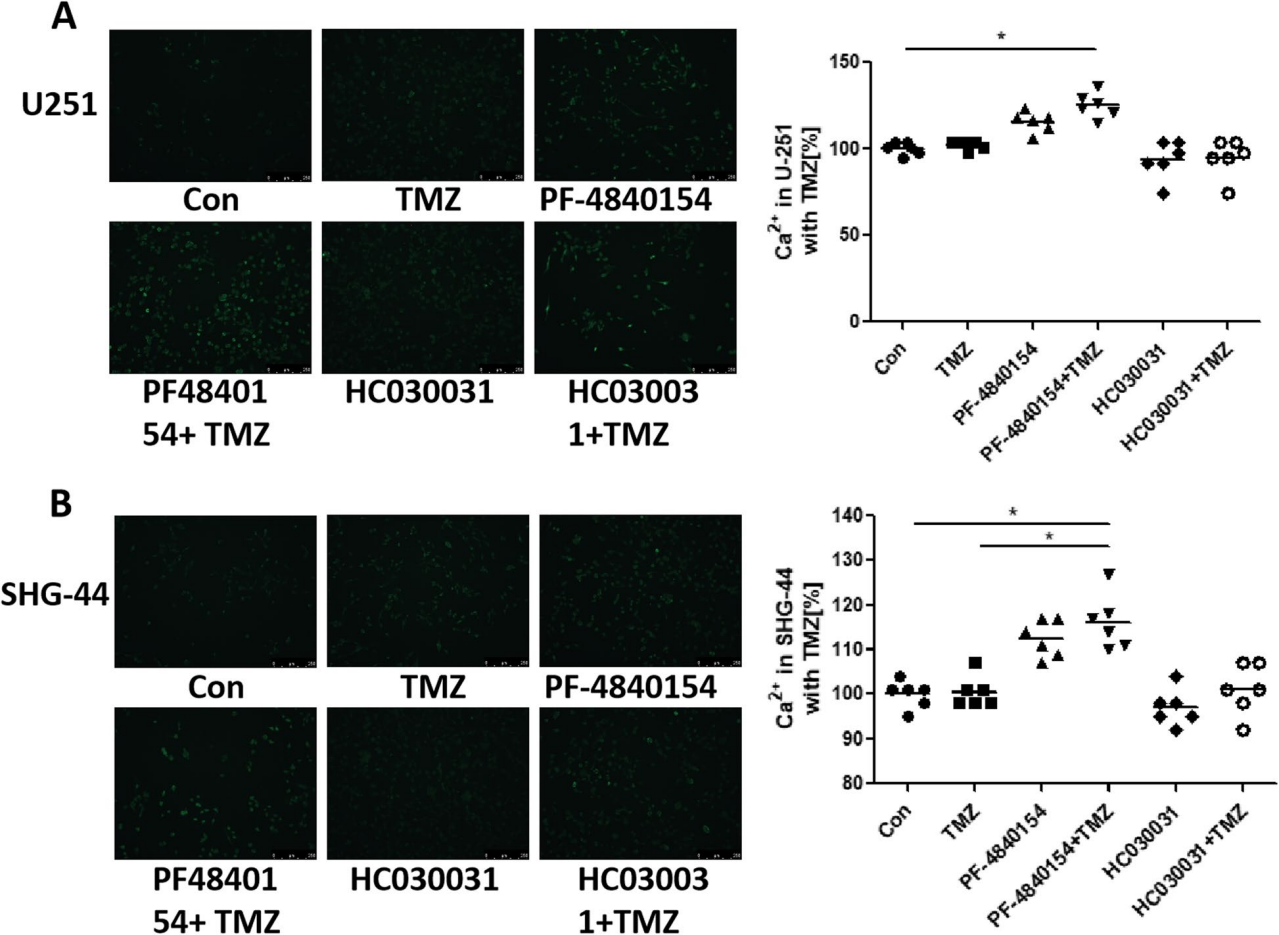
Effect of TMZ and a TRPA1 agonist inhibitor on intracellular Ca^2^+ levels. The levels of intracellular Ca^2^+ were increased by exposure to TMZ and PF-4840154 cotreatment, as determined by fluorescence microscopy using a Flexstation®2 using a Fluo-4 A.M. in U251 cells (**A**) and SHG-44 cells (**B**). Representative microscopy images are shown (100 x) with mean values and data from individual plate reader experiments reported graphically below. *P < 0.05, **P < 0.01, ***P < 0.001, ****P < 0.0001

Since TRPA1 is an important calcium channel, when the channel is open, a large amount of calcium ions enter cells to initiate downstream reactions. Therefore, we considered that the relationship between TRPA1 and TMZ resistance in GBM cells is related to TRPA1 signaling. To further explore the mechanism by which phenotypes are acquired, we used a fluorescent probe to determine whether the intracellular Ca^2+^ level was influenced by TMZ treatment or TRPA1 disruption. Representative images are shown in Fig. 3. The figures show that the U251 cells in the PF-4840154 group and TMZ and PF-4840154 cotreatment group exhibited slightly increased intracellular Ca^2+^ compared to that in the other groups (Fig. 4A). Additionally, in SHG-44 cells, intracellular Ca^2+^ was significantly increased in the TMZ and PF-4840154 cotreatment group compared to the TMZ group (Fig. 4B).

### TRPA1 activation and TMZ treatment induced mitochondrial dysfunction

**Fig. 5:**
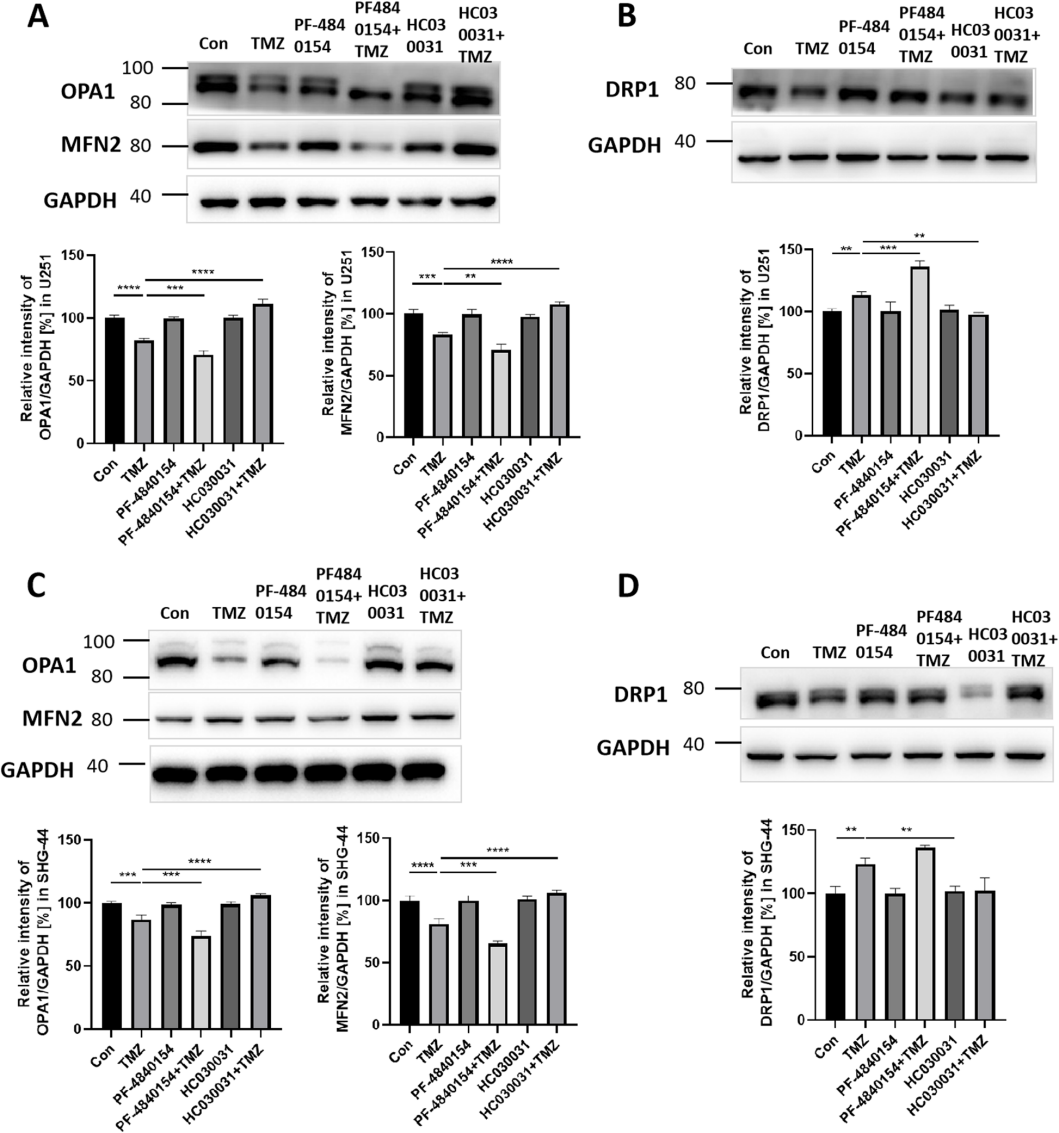
Effect of TMZ and a TRPA1 agonist and inhibitor on mitochondrial fission and fusion protein expression. Western blot assay showing the levels of the mitochondrial fusion proteins optic atrophy 1 (OPA1) and Mitofusin 2 (MFN2) (**A**) and the mitochondrial fission protein dystrophin-related protein 1 (DRP1) (**B**) in U251 cells after treatment with TMZ and a TRPA1 agonist and inhibitor. Western blot assay showing the levels of OPA1, MFN2 (**C**), and DRP1 (**D**) in SHG-44 cells after treatment with TMZ and a TRPA1 agonist and inhibitor. GAPDH was used as an internal reference. Cropped blots are displayed, and the expression level was quantified by densitometry with ImageJ software. *P < 0.05, **P < 0.01, ***P < 0.001, ****P < 0.0001

Mitochondria are the main sources of ROS in cells and may be involved in oxidative stress resistance. To investigate whether mitochondrial dynamics were disrupted by activating TRPA1 and triggering Ca^2+^ influx, mitochondrial fission and fusion protein expression was determined by WB analysis. Representative pictures are shown in Fig. 5. Compared with that in the control and TMZ groups, U251 cells pretreated with PF-4840154 decreased the expression of the mitochondrial fusion proteins OPA1 and MFN2 and increased the expression of the mitochondrial fission protein DRP1 (Fig. 5A and B). The results showed that the mitochondrial structure was damaged. In addition, compared to the TMZ group, the TMZ and HC030031 cotreatment group showed that the expression of OPA1 and MFN2 was increased and that the expression of DRP1 was decreased (Fig. 5A and B). The SHG-44 cells showed effects similar to those observed with the U251 cells (Fig. 5C and D). All original western blot images have been included in Supplementary original western blot images 2.

Our results suggest that the damage to mitochondrial structure caused by changes in TRPA1 expression may be critical to the development of oxidative stress resistance in tumor cells.

### Overexpression of TRPA1 induced cell apoptosis and ROS production via increased intracellular Ca2+ levels in TMZ-stimulated U251 cells

Since the use of agonists alone to increase TRPA1 expression leads to uncontrollable outcomes, with no way to modulate the effects, side effects may occur. Therefore, we used TRPA1-overexpressing and shRNA U251 cells to directly observe changes in tumor cells when TRPA1 expression is increased.

**Fig. 6:**
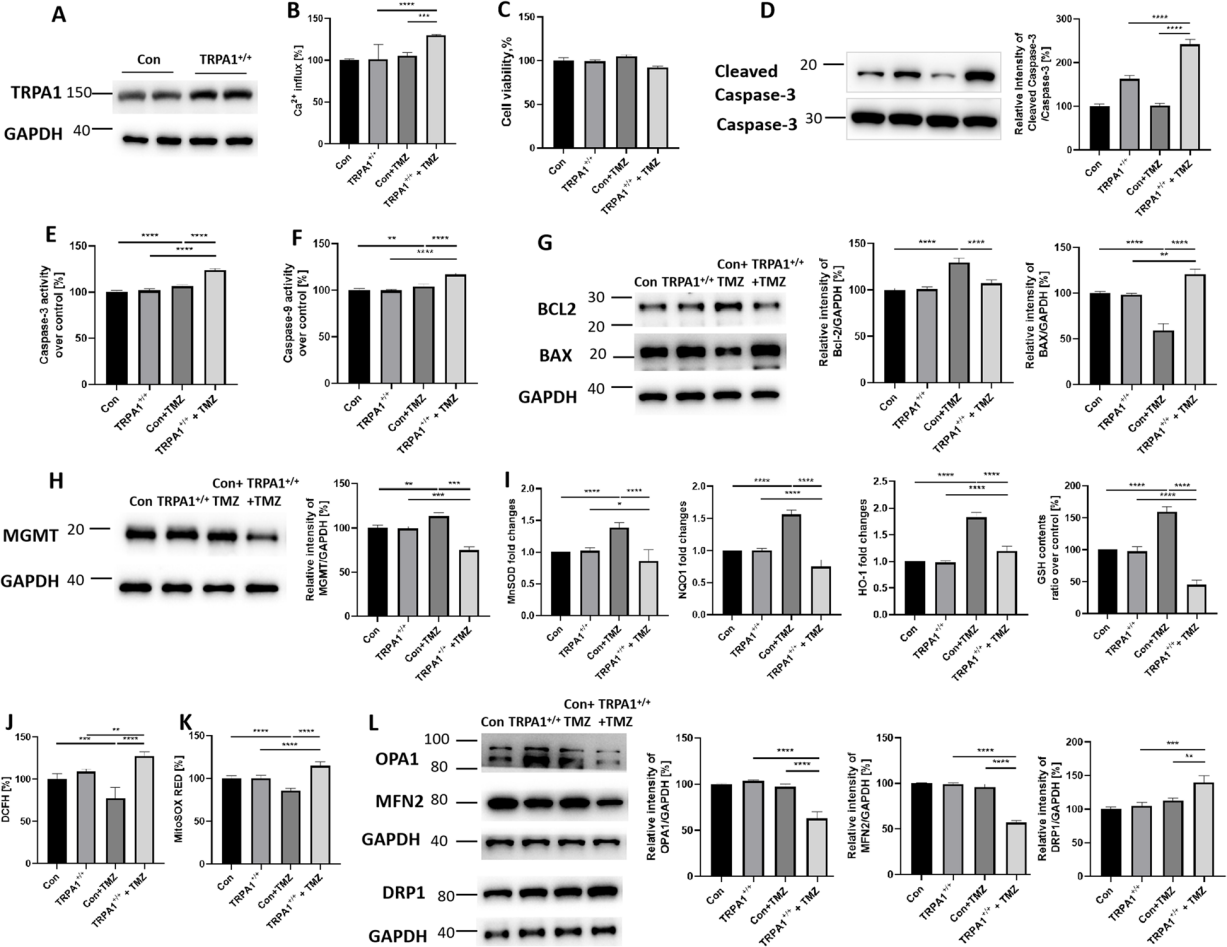
Overexpression of TRPA1 Induced Cell Apoptosis and ROS Production via Increased Intracellular Ca^2+^ Levels in TMZ-Stimulated U251 Cells. **A** Transfection of U251 cells with TRPA1 shRNA upregulated (TRPA1^+/+^) TRPA1 protein expression at 12 h compared with control shRNA (Con) treatment. The cropped blots are displayed. **B** The level of Ca^2+^ influx changed. TMZ stimulation showed different effects on cell viability (**C**), the expression of the Cleaved Caspase-3 protein (**D**), the activity of Caspase-3 (**E**) and Caspase-9 (**F**), and the level of apoptosis proteins (**G**) in Con and TRPA1^+/+^ U251 cells. Additionally, the effects of TMZ on the protein expression of MGMT (**H**), the levels of the cell antioxidant genes MnSOD, NQO1 and HO-1, and antioxidants GSH (**I**). intracellular ROS (**J**) and mitochondrial ROS (**K**), as well as the levels of mitochondrial structural proteins (**L**) in Con and TRPA1^+/+^ U251 cells. GAPDH was used as an internal reference. The cropped blots were displayed, and the expression level was quantified densitometry with ImageJ software. *P < 0.05, **P < 0.01, ***P < 0.001, ****P < 0.0001

As shown in Fig. 6A, the level of TRPA1 was significantly increased in the TRPA1^+/+^ cells (TRPA1^+/+^) compared to the control cells (Con). The increased expression of TRPA1 also augmented the level of Ca^2+^ influx in TMZ-stimulated U251 cells (Fig. 6B). TMZ administration led to no obvious change in cell viability but increased the activity of Caspase-3 and Caspase-9 in both the control cells and TRPA1^+/+^ cells. Moreover, the level of Cleaved Caspase-3 protein, the Caspase-3 and Caspase-9 activity was increased in the TRPA1^+/+^+TMZ group compared to the Con+TMZ group (Fig. 6C, D, E and F). As shown in Fig. 6G, the WB results indicated that Bcl2 expression was increased and BAX expression was decreased in Con+TMZ groups. However, the comparison between the Con+TMZ and the TRPA1^+/+^+TMZ groups showed that TMZ clearly decreased the level of Bcl2 and increased the level of BAX in the latter group. All original western blot images have been included in Supplementary original western blot images 3. These results indicated that increasing the expression of TRPA1 induced an increase in intracellular Ca^2+^ and then significantly increased the tumor killing effect of TMZ.

Consistent with the agonist effects, TMZ stimulation increased the expression of MGMT in the control cells. In the TRPA1^+/+^ cells, TMZ stimulation decreased the expression levels of MGMT (Fig. 6H). Also TMZ stimulation increased the expression of MnSOD, NQO1, HO-1 and GSH in the control cells. In the TRPA1^+/+^ cells, TMZ stimulation decreased the expression levels of antioxidant genes and GSH (Fig. 6I). Corresponding to this result, the results of the DCFH-DA and MitoSOX Red assays also showed that TMZ induced a decrease in intracellular ROS and mitochondrial ROS levels in the control cells but an increase in these levels in the TRPA1^+/+^ cells (Fig. 6J and K). In control cells, TMZ stimulation led to no obvious changes in the levels of mitochondrial fission or fusion protein expression. However, the expression levels of the mitochondrial fusion proteins OPA1 and MFN2 were decreased, and the expression of the mitochondrial fission protein DRP1 was increased in TMZ-stimulated TRPA1^+/+^ cells (Fig. 6L). These results indicate that the elevated expression of TRPA1 is involved in TMZ-induced mitochondrial structural damage, which leads to the accumulation of mitochondrial ROS and intracellular ROS in tumor cells and ultimately increases the apoptosis rate.

## Discussion

In this study, we demonstrated that upregulating TRPA1 overwhelmed TMZ resistance in GBM cells by disrupting the mitochondrial dynamics of cells, and the role played by TRPA1 may lead to increased calcium influx.

In U251 and SHG-44 cells, TMZ induced only slightly increased the apoptosis rate and intracellular and mitochondrial ROS levels. In addition, TMZ induced increased MGMT protein and antioxidants expression in these cells. Pretreatment with a TRPA1 agonist significantly decreased the level of MGMT protein and antioxidants expression; enhanced intracellular and mitochondrial ROS levels; induced Ca^2+^ influx, mitochondrial damage and cell apoptosis; and disrupted the balance between mitochondrial fission and fusion proteins in GBM cells. Pretreatment with a TRPA1 inhibitor slightly enhanced the level of MGMT protein and antioxidants expression, and reduced the apoptosis rate. These results suggest that the TRPA1 pathway plays an important role in reducing the TMZ resistance of GBM cells. To eliminate the side effects of the administered drugs, we exposed U251 cells to overexpressed shRNA and TRPA1. The results were similar to those of the agonist treatment. However, the decrease in TRPA1^+/+^ cell viability induced by TMZ was not statistically significant compared to that in control cells. However, the increased expression of TRPA1 led to a significant increase in apoptosis indicators, including increased the level of Cleaved Caspase-3 protein, as well as the caspase-3 and caspase-9 activity, as well as decreased expression of MGMT protein, the anti-apoptosis protein Bcl2 and increased expression of the apoptosis protein BAX. Additionally, damage to mitochondrial structures induced mitochondrial dysfunction. Therefore, both the mitochondrial and intracellular oxidative stress responses were significantly increased, and the oxidative stress resistance of the tumor cells was eventually diminished.

The TRP superfamily is composed of cation-selective channels that play key roles in sensory physiology, such as thermo- and osmosensation, as well as in several diseases, such as cardiovascular, cancer and neuronal diseases [31]. Deveci et al. [32] showed that the apoptotic, inflammatory and oxidant effects of hypoxia were increased due to the activation of TRPA1. In the present study, we observed increased rates of apoptosis, greater Ca^2+^ influx and higher intracellular ROS levels after activation of the TRPA1 channels in GBM cells. Moreover, the activation of TRPA1 increased mitochondrial oxidative stress and mitochondrial dysfunction. Therefore, the oxidative stress response leading to the apoptosis of tumor cells can be induced through TMZ stimulation.

TRPA1 can be activated in neurons and cancer cells through different stimuli [27, 28]. In cancer cells, the activation of TRPA1 channels increases Ca^2+^ influx, cell migration, and, presumably, tumor cell invasion [29]. Moreover, because of TRPA1 activation, the Ca^2+^-dependent antiapoptotic pathways are activated [30]. This finding pertains to tumor cells because TRP channels can be targets of therapeutic agents, contributing to inhibited tumor growth and increased tumor-induced inflammation [29]. In addition, TRPA1 activation is increased by an increase in mitochondrial ROS production, and GBM cells are killed by the TRPA1 channel-induced excessive production of intracellular ROS, apoptosis and Ca^2+^ entry [32].

Our results showed that the TRPA1 pathway caused increased intracellular Ca^2+^ levels via ion influx. In U251 and SHG-44 cells, pretreatment with PF-4840154 increased the intracellular Ca^2+^ level and induced cell apoptosis. In addition, blocking TRPA1 activity prevented Ca^2+^ influx and decreased cell apoptosis. Previous studies have reported that Ca^2+^ signaling mediated by various Ca^2+^ channels regulates the activity of cellular NADPH oxidase, which in turn results in an elevation in intracellular ROS levels [33]. These results proved that the Ca^2+^ level in cells may be related to the apoptosis rate of GBM cells.

In the present study, TMZ alone failed to elevate the intracellular and mitochondrial ROS levels, but activating TRPA1 increased the oxidative stress reaction in GBM cells. When U251 and SHG-44 cells were cotreated with TMZ and PF-4840154, the intracellular and mitochondrial ROS levels in the cells significantly increased compared to those after treatment with TMZ alone. The overexpression of TRPA1 in U251 cells confirmed these results. The increased oxidative reaction in cells led to subsequent cell damage and apoptosis. These results were similar to that of a previous study in which activating the TRPA1 channel induced apoptosis and inhibited cell survival. Ca^2+^ has been reported to be transferred from the cytosol during mitochondrial stress to the mitochondria and mediates excessive ROS generation [34]. Accroding to Previous studies, The MGMT expression is an important determinant of TMZ resistance, the modification of the level of MGMT protein by its transcription factors has been proposed as a method to sensitize tumors to TMZ. Thus, the downregulation of MGMT is considered a good prognostic factor and results in a longer survival period in GBM patients treated with TMZ [35]. Our results also demonstrated that up-regulated TRPA1 expression could decreased the level of MGMT, which was related to the TMZ resistance of GBM cells.

TMZ increased the expression of antioxidant gene mRNA levels in both the U251 and SHG-44 cell lines. In the U251 cells, pretreatment with PF-4840154 or overexpression of the TRPA1 gene effectively reduced TMZ-enhanced antioxidant expression, and the expression of MnSOD, NQO1, HO-1 and GSH was decreased. However, pretreatment with HC030031 did not lead to obvious changes. In the SHG-44 cells, PF-4840154 induced a decrease in antioxidant gene expression. PF-4840154 treatment alone reduced the expression of MnSOD and NQO1, while PF-4840154 and TMZ cotreatment significantly exacerbated the reduction in MnSOD, NQO1, HO-1 and GSH expression. These results confirmed the role played by the TRPA1 pathway in regulating the cellular oxidative stress response. In GBM cells, TMZ treatment induced an increase in cellular reactive antioxidants, which reduced the extent of cell damage and rate of apoptosis. Activation of the TRPA1 pathway increased the influx of Ca^2+^ and reduced the release of antioxidants, ultimately inducing oxidative stress damage in cells.

Increasing mitochondrial stress and mitochondrial dysfunction through activation of TRPA1 has been suggested to account for apoptosis induction of cancer cells [17]. Mitochondrial structure is important in controlling mitochondrial function [36]. Our data showed that TMZ slightly influenced the mitochondrial structure in both GBM cell lines. The present study also demonstrated that a TRPA1 agonist destroyed the balance between mitochondrial fission and fusion protein expression in both cell lines. Pretreatment with PF-4840154 reduced the expression of mitochondrial fusion proteins and increased the expression of mitochondrial fission proteins. The expression level of fission/fusion protein led to imbalances that contributed to mitochondrial dysfunction, permeabilization of the outer mitochondrial membrane, and apoptotic protein release, ultimately promoting cell death [37].

We posited a scenario in which GBM cells generate ROS resistance to TMZ treatment, thereby reducing the therapeutic effect of TMZ. Activation of the TRPA1 pathway induces Ca^2+^ influx and oxidative stress. Mitochondrial dysfunction induces intracellular and mitochondrial ROS levels which trigger in increase in cell apoptosis.

Intracellular Ca^2+^, a secondary messenger, regulates gene transcription, cell proliferation and migration, and cell death. The increase in intracellular Ca^2+^ and ROS levels induces the molecular cascade leading to apoptosis. Finally, the nucleic acid, protein, and lipid substances in the cell structure are transformed due to increased oxidative stress and mitochondrial depolarization [38–40].

Our study highlighted the importance played by the TRPA1 pathway in regulating GBM cell apoptosis in association with oxidative stress. Targeting the TRPA1 pathway is a promising novel approach to GBM treatment that may show the benefits of reducing GBM resistance to TMZ and increasing drug sensitivity and the apoptosis rate.

## Supplementary Information

**Additional file 1.**

## Data Availability

All data generated or analysed during this study are included in this published article.

## Abbreviations

GBM: Glioblastoma
TMZ: Temozolomide
ROS: Reactive oxygen species
TRP: Transient receptor potential
MFN: Mitofusion
OPA: Optic atrophy
FIS: Fission1
DRP: Dynamin-related protein
MFF: Mitochondrial fission factor
