# Supplementary material for: Activated TRPA1 plays a therapeutic role in TMZ resistance in glioblastoma by altering mitochondrial dynamics
Source: BMC Mol Cell Biol. 2022 Aug 19;23:38. doi: 10.1186/s12860-022-00438-1 (PMC9389719; doi:10.1186/s12860-022-00438-1)
Supplement: Supplementary file 1 — Additional file 1. [file 12860_2022_438_MOESM1_ESM.doc]

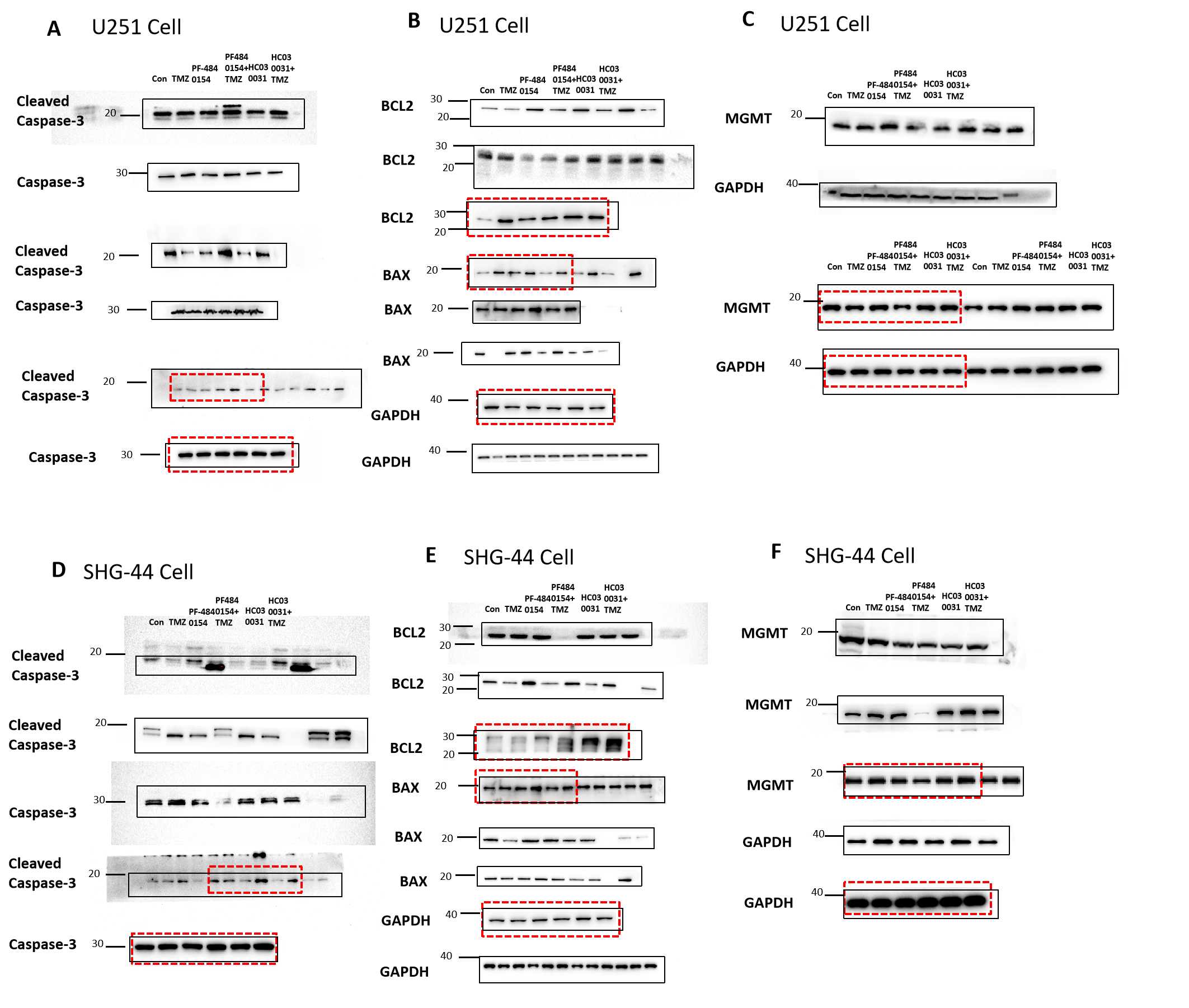


**Supplementary original western blot images 1. Original blots and replicate experimental images corresponding to all the images in Fig. 2.**

All Western blot images were showed. We outlined the edges of the blots using solid black lines, and the regions of the original blots used in main figures were denoted using red boxes. (A) All western blot images of Cleaved Caspase-3 and Caspase-3 proteins in U251 cells. (B) All western blot images of BCL2, BAX and GAPDH proteins in U251 cells. (C) All western blot images of MGMT and GAPDH proteins in U251 cells. (D) All western blot images of Cleaved Caspase-3 and Caspase-3 proteins in SHG-44 cells. (E) All western blot images of BCL2, BAX and GAPDH proteins in SHG-44 cells. (F) All western blot images of MGMT and GAPDH proteins in SHG-44 cells. The membrane was tailored according to the molecular weight of the target protein before incubated with primary antibodies.


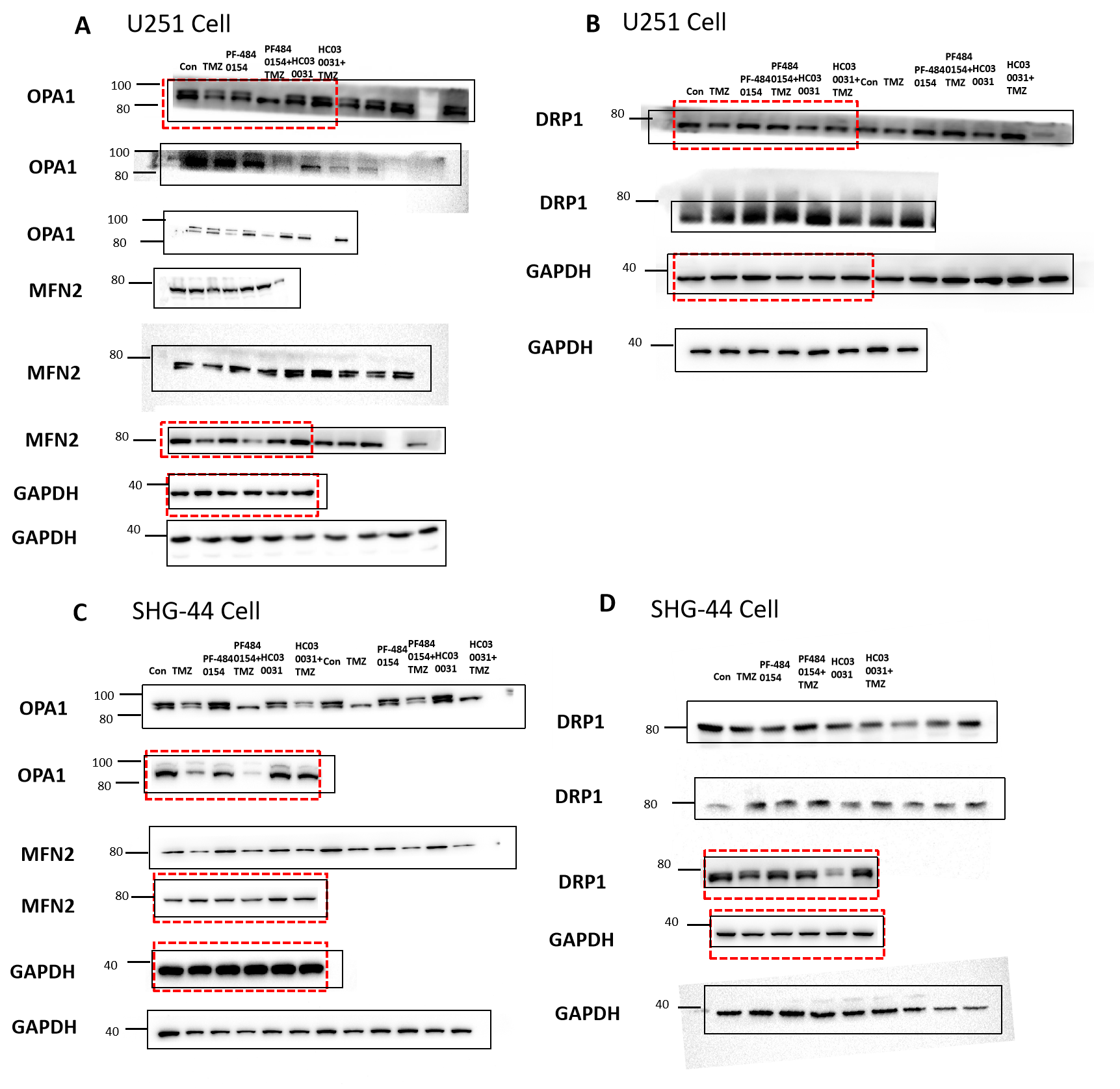


**Supplementary original western blot images 2. Original blots and replicate experimental images corresponding to all the images in Fig. 5.**

All Western blot images were showed. We outlined the edges of the blots using solid black lines, and the regions of the original blots used in main figures were denoted using red boxes. (A) All western blot images of OPA1, MFN2 and GAPDH proteins in U251 cells. (B) All western blot images of DRP1 and GAPDH proteins in U251 cells. (C) All western blot images of OPA1, MFN2 and GAPDH proteins in SHG-44 cells. (D) All western blot images of DRP1 and GAPDH proteins in SHG-44 cells. The membrane was tailored according to the molecular weight of the target protein before incubated with primary antibodies.


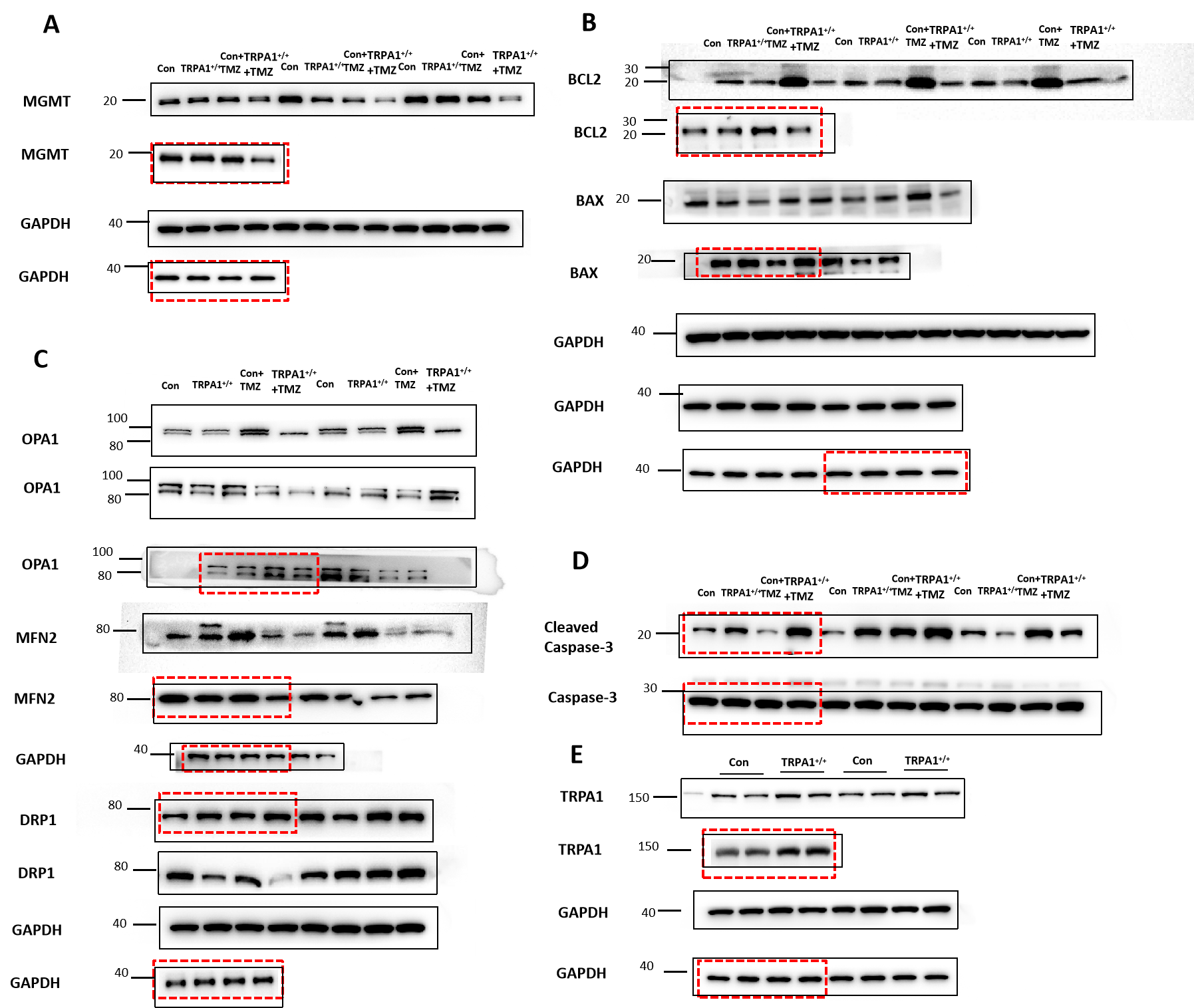


**Supplementary original western blot images 3. Original blots and replicate experimental images corresponding to all the images in Fig. 6.**

All Western blot images were showed. We outlined the edges of the blots using solid black lines, and the regions of the original blots used in main figures were denoted using red boxes. (A) All western blot images of MGMT and GAPDH proteins in U251 cells. (B) All western blot images of BCL2, BAX and GAPDH proteins in U251 cells. (C) All western blot images of OPA1, MFN2, DRP1 and GAPDH proteins in U251 cells. (D) All western blot images of Cleaved Caspase-3 and Caspase-3 proteins in U251 cells. (E) All western blot images of TRPA1 and GAPDH proteins in U251 cells. The membrane was tailored according to the molecular weight of the target protein before incubated with primary antibodies.
